# A minimum of two distinct heritable factors are required to explain correlation structures in proliferating lymphocytes

**DOI:** 10.1098/rsif.2009.0488

**Published:** 2010-01-06

**Authors:** John F. Markham, Cameron J. Wellard, Edwin D. Hawkins, Ken R. Duffy, Philip D. Hodgkin

**Affiliations:** 1 Department of Electrical and Electronic Engineering, Victorian Research Laboratory, National ICT Australia, The University of Melbourne, Victoria 3010, Australia; 2 Immunology Division, The Walter and Eliza Hall Institute of Medical Research, 1G Royal Parade, Victoria 3050, Australia; 3 Immune Signalling Laboratory, Cancer Immunology Research Program, Peter MacCallum Cancer Institute, St Andrews Place, East Melbourne, Victoria 3002, Australia; 4 Hamilton Institute, National University of Ireland Maynooth, Ireland

**Keywords:** cell lifespan, cell proliferation, cell division, mathematical model, immune response

## Abstract

During the adaptive immune response, lymphocyte populations undergo a characteristic three-phase process: expansion through a series of cell divisions; cessation of expansion; and, finally, most of the accumulated lymphocytes die by apoptosis. The data used, thus far, to inform understanding of these processes, both *in vitro* and *in vivo*, are taken from flow cytometry experiments. One significant drawback of flow cytometry is that individual cells cannot be tracked, so that it is not possible to investigate interdependencies in the fate of cells within a family tree. This deficit in experimental information has recently been overcome by Hawkins *et al*. (Hawkins *et al*. 2009 *Proc. Natl Acad. Sci. USA* **106**, 13 457–13 462 (doi:10.1073/pnas.0905629106)), who reported on time-lapse microscopy experiments in which B-cells were stimulated through the TLR-9 receptor. Cells stimulated in this way do not aggregate, so that data regarding family trees can be recorded. In this article, we further investigate the Hawkins *et al*. data. Our conclusions are striking: in order to explain the familial correlation structure in division times, death times and propensity to divide, a minimum of two distinct heritable factors are necessary. As the data show that two distinct factors are necessary, we develop a stochastic model that has two heritable factors and demonstrate that it can reproduce the key features of the data. This model shows that two heritable factors are sufficient. These deductions have a clear impact upon biological understanding of the adaptive immune response. They also necessitate changes to the fundamental premises behind the tools developed by statisticians to draw deductions from flow cytometry data. Finally, they affect the mathematical modelling paradigms that are used to study these systems, as these are widely developed based on assumptions of cellular independence that are not accurate.

## 1. Introduction

The reciprocal cellular processes of division and apoptosis combine to regulate biological processes ranging from patterning body and tissue shape to regulation and maintenance of the numbers of red blood cells, platelets, monocytes and lymphocytes in the blood. As a result of the ubiquity of this mechanism, there is tremendous general interest in the regulation and simultaneous control of division and death. Investigators, from as early as the 1950s, have used film and microscopy to observe and measure the kinetics of cell division *in vitro* (Powell 1955; Dawson *et al*. 1965; Minor & Smith 1974; Collyn-D'Hooghe *et al*. 1977; Absher & Cristofalo 1984). These studies, on a variety of cell types, all report that inter-mitotic division times show significant variation within clones of growing cells. Both quantitative and qualitative explanations were given to describe this variation. The influential Smith and Martin model proposed that variation originated from a stochastic regulator operating in an ‘A state’ (assumed to be G1) that governed entry into a deterministic B phase (S, G2 and M) of the cell cycle (Smith & Martin 1973). Alternatively, size models implicated imprecise inheritance of cellular components regulating growth and replication as being responsible for differences in division times (Clifford & Sudbury 1972; Tyson & Diekmann 1986). The source of the interdivisional variation or its significance is still not known. Cells undergoing apoptosis also show variation in times to die that are consistent with a stochastic internal process that is at least partly the result of a balance of anti- and pro-apoptotic molecules (Hawkins *et al*. 2007; Spencer *et al*. 2009). Similarly, little is understood about how control of division and apoptosis is related and how this relation affects control of cell populations in an immune response.

An excellent system for studying complex population shaping by regulated division and death is the adaptive immune response mounted by both T and B lymphocytes. At its core is the clonal expansion of lymphocytes of given specificity owing to the appearance of antigen. During this response, B- and T-cell populations undergo a characteristic three-phase process: expansion through a series of cell divisions; cessation of expansion; and, finally, most of the accumulated lymphocytes die by apoptosis. Advances in flow cytometry and the discovery of non-interfering fluorescent dyes that act as cell labels have enabled the collection of experimental data on the kinetics of lymphocyte division progression and cell survival (e.g. Lyons & Parish 1994; Parish 1999). These techniques yield high-quality information at the level of populations. For example, use of the fluorescent dye carboxyfluorescein succinimidyl ester (CFSE) can provide a time course for the number of live and dead lymphocytes and the fraction of cells that have undergone any given number of cell divisions. These data have strongly influenced immunological understanding. They have inspired statisticians to develop methodologies to study flow cytometry data (e.g. Hyrien & Zand 2008), and provided information on which modellers have based their paradigms (e.g. Gett & Hodgkin 2000; Leon *et al*. 2004; Ganusov *et al*. 2005; Hawkins *et al*. 2007).

Data from these experiments are not, however, without their limitations. One significant drawback of flow cytometry data is that individual cells cannot be tracked, so that it is not possible to investigate dependencies in the fate of cells within a family tree. In the absence of this information, biologists, statisticians and modellers assume that all cells act as independent entities. This deficit in experimental information has recently been overcome by Hawkins *et al*. (2009), who reported on time-lapse microscopy experiments in which B-cells were stimulated through the toll-like receptor-9 (TLR-9) receptor. Cells stimulated in this way undergo the usual population dynamics, dividing for 2–6 generations, but do not aggregate, so that extensive data regarding family trees can be observed and recorded.

In this article, we detail a further investigation of the Hawkins *et al*. (2009) data. In order to explain the familial correlation structure in division times, death times and propensity to divide, a minimum of two distinct heritable factors is necessary. One factor regulates the propensity for a cell to divide and, if it does so, the time at which it divides. The other factor relates the propensity for cell division and the time taken to apoptosis. We then develop a stochastic model that has two heritable factors and demonstrate that it can reproduce the key features of the data. Thus, the data show that two distinct factors are necessary and the model shows that two are also sufficient. These deductions have important implications for mathematical modelling paradigms that are used to study these systems.

## 2. Results

### 2.1. The B-cell dataset

Hawkins *et al*. (2009) have reported a dataset derived from visual annotation of dividing primary naive B lymphocytes stimulated using the TLR-9 ligand, CpG. The initial populations of cells and their progeny were cultured on Terasaki plates and followed for 120 h using time-lapse microscopy. Images were taken of the cells in seven of the wells in each plate at a frequency of one per 2 min. Cell division was judged manually and cell death was judged by manual observation of propidium iodide uptake as a result of loss of membrane integrity upon apoptosis. Pedigrees of cells were followed from stimulation for up to seven rounds of division, by which time nearly all cells had died. In total, 107 and 89 pedigrees were followed in two different experiments (Fam2 and Fam3, respectively) and times to die and divide for related cells recorded.

The data presented by Hawkins *et al*. (2009) are the first available for primary lymphocytes and the first individual cell tracking experiments to include substantial information concerning cell death times and division cessation. They noted a number of trends in the average behaviour of the cell population that gave some insight into cell operation and particularly the extent of inheritance. We first summarize these features and then report on new correlations that must be accommodated into any description of cellular inheritance. Then, we present a physical model with a demonstrably minimum number of heritable factors that has the ability to reproduce these features.

### 2.2. Trends and correlations in the dataset

**Figure 1.**
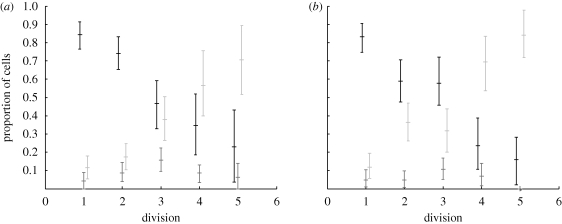
Trends in cell fate broken down per division for experiments (*a*) Fam2 and (*b*) Fam3. In both cases, the proportion of siblings undergoing different fates is a maximum mid-response. Black, both siblings divide; dark grey, one sibling divides; light grey, both siblings die.

CpG-stimulated naive B-cells typically undergo a series of between one and six division rounds. The time to first division takes approximately 35 h while the more rapid subsequent divisions average 10 h, although the mean time increases by approximately 10–15% in the later division rounds. As noted for many other cell types, the times to divide are highly variable and, when plotted as a frequency histogram, follow a right skewed distribution. Hawkins *et al*. (2009) also noted a high degree of correlation in siblings’ division times. Other reported features included the phenomenon of division destiny where cells ceased to both grow and divide after 2–6 division rounds. These cells eventually died with the times to die also highly variable, with the mean time decreasing by approximately 25–35% in the later division rounds. The division destiny of progeny was heritable and strongly dependent on the original founder cell which Hawkins *et al*. (2009) illustrated using a heat map to display division destiny of cell pedigrees. This effect comes about because the fate of siblings cells (that is, whether they divide or die) is highly correlated. Figure 1 presents a new quantitative representation of this relationship. The fate of siblings is broken down per division. In early divisions, it is almost always observed that both siblings divide, while in later divisions it is almost always the case that neither sibling divides. Only in the middle phase of the response do we find siblings having different responses and even then this is in less than 20 per cent of cases.

**Figure 2.**
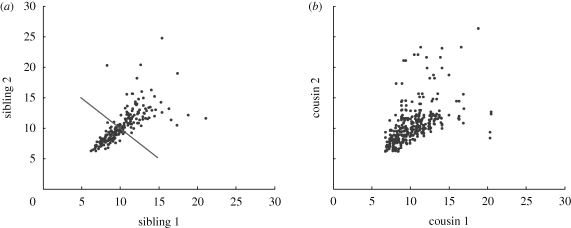
Correlation of times to divide for related cells (Fam2). (*a*) Times to divide for siblings are more highly correlated than for first cousins. (*b*) Siblings whose division times sum to more than 20 h (above and to the right of the solid line in (*a*)) are less correlated (*r* = 0.23) than the population as a whole (*r* = 0.71). (*a*) *r* = 0.71, *n* = 204; (*b*) *r* = 0.50, *n* = 352.

**Figure 3.**
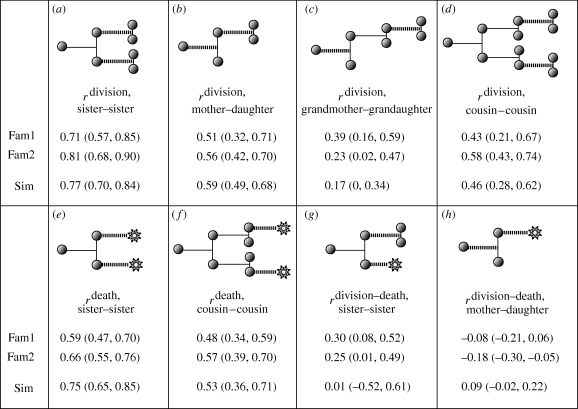
Measured correlation (Pearson *r*) from experiment compared with simulation. Illustration shows the correlations being measured by heavy dashed lines. Figures within brackets show the 95% confidence interval obtained using a bootstrapping method as described in the electronic supplementary material.

Figure 2 presents the correlations in division times for siblings and first cousins for one experimental set of results labelled Fam2 (the other data are qualitatively similar). Each is positively correlated (figure 3*e*,*f*). In order to check that this correlation is not simply due to the dependence of time to divide on number of divisions, we looked at the correlations in subpopulations of cells which had undergone an identical number of divisions and found the same result (data not shown). Interestingly, the correlation between sibling times to divide is particularly strong at earlier division times as can be seen in figure 2*a*, where, if we exclude siblings whose division times sum to less than 20 h, the Pearson correlation coefficient (Sokal & Rohlf 1995), *r*, falls from 0.71 to 0.23.

### 2.3. Propensity to divide of related cells is strongly correlated

In the following discussion, we use the term ‘propensity to divide’ to describe the likelihood of a cell to divide. In this experiment, four different cell outcomes are observed: cells can be observed to undergo division or death, cells can be lost from view (around 17%) and a small number (2.5%) reach the end of the experiment alive. We assume that, after sufficient time has elapsed, all cells will undergo one of two fates, division or death. We measure the correlation of fates of sibling cells by assigning the number 1 to division and 0 to death and measuring Pearson's *r* for these numbers. So, for example, if siblings always had the same fate (that is, if one divided then the other always divided or vice versa), then they would have *r* = 1. Conversely, if the fate of sibling cells was independent, then they would be uncorrelated and have *r* = 0. After doing so, we find that, according to this method, cell fate is strongly correlated between siblings and equal to 0.81 (0.76, 0.86) for Fam2 and 0.87 (0.82, 0.91) for Fam3 (95% confidence intervals in brackets). It is also a heritable property, as demonstrated by the correlation between cousins' propensity to divide and also the clonal property whereby all cells in a clone lose their impetus to divide after approximately the same number of divisions (Hawkins *et al.* 2009).

### 2.4. Propensity to divide is correlated to both time to divide and time to die

**Figure 4.**
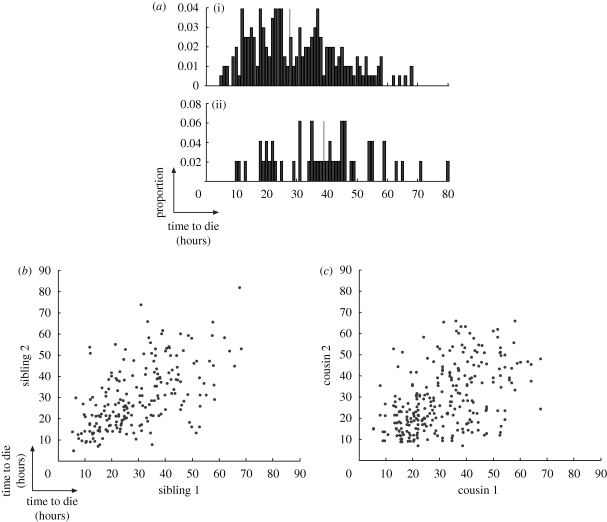
Correlation of times to die for related cells (Fam2). (*a*) When both siblings die, they tend to do so earlier than cells whose siblings divide (*p* < 0.0001 for median value; see the electronic supplementary material for details). (*a*)(i) Time to die given other sibling died, median = 27.7 h; (*a*)(ii) time to die given other sibling divided, median = 39 h, *n* = 49. (*b*) Times to die for sisters are more highly correlated than for (*c*) cousins. (*b*) *r* = 0.57, *n* = 203; (*c*) *r* = 0.47, *n* = 267.

In Hawkins *et al*. (2009), it was shown that a heritable factor both increases the propensity to divide and shortens the time to divide. Here, we find a correlation between propensity to divide and time to *die*. Figure 4*a* illustrates this by showing that cells whose siblings divide tend to die later than cells whose siblings die. These data lead us to conclude that a common factor influences both time to die and propensity to divide. The observation that time to die is correlated between siblings and cousins (figure 4*b*,*c*) suggests that such a factor is heritable.

### 2.5. One common factor is not sufficient to describe the data

**Figure 5.**
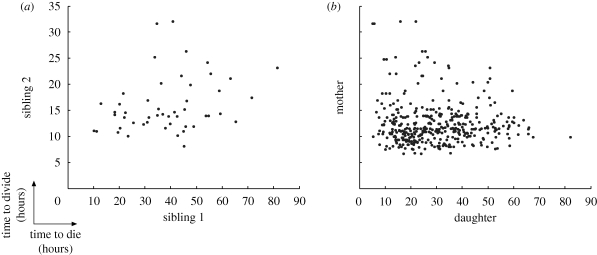
Correlation between times to divide and die (Fam2). (*a*) Sisters which undergo different fates show a small amount of positive correlation between their respective times to die and divide: *r* = 0.29, *n* = 49. (*b*) There is no significant correlation (refer to figure 3*g*) between when a cell divides and when its offspring die: *r* = −0.07, *n* = 384.

If the putative common factor that regulates propensity to divide and time to divide is the same as the common factor that regulates propensity to divide and time to die, then one might expect to observe a consistent negative correlation between time to die and time to divide for related cells. We looked for this in two places. First of all, there is a small subset of sibling cells which undergo different fates. Data from such siblings (figures 5*a* and 3*g*) show that there is a small positive correlation although, as mentioned, the number of sibling cells with uneven fates is small. Secondly, we looked at the relationship between mother time to divide and daughter time to die (figure 5*b*) and found no significant correlation (see also figure 3*h*). This is strongly suggestive that at least two independent heritable factors are necessary to explain the data.

### 2.6. Modelling division times

Having established that at least two heritable factors are necessary to explain the data, we now demonstrate that two are sufficient for a mathematical model to reproduce the most significant features of the data. The features of the data can be divided into three categories: statistics describing division times, statistics describing death times and those describing fate determination. Our approach will be to work through each category in turn, developing a minimal model that can describe all the features in each category. At the end of the process, we will have a unified, minimal model that can describe the relevant features of the data using two heritable factors. Our test for sufficiency will be to identify the important criteria in the various aspects of measured cell behaviour and to show how our two-factor model can satisfy each one.

We start by looking at division times. Based on the above discussion, we seek a model that can reproduce the following features of the data.

— Right skewed distribution with a minimum division time of approximately 6 h.
— A trend of increasing *t*_divide_ as a function of generations.
— Correlated *t*_divide_ for siblings and inheritance of *t*_divide_ from mother cells.
— Correlation of *t*_divide_ for siblings being stronger for pairs of cells that divide earlier.

We found that models that divided the cell cycle into a series of steps with deterministic and exponential waiting times, such as the Smith–Martin transition probability model (Smith & Martin 1973), had difficulty reproducing the experimentally observed strong correlations at early division times (data not shown). In contrast, we will show that a development of the modelling framework first proposed as Castor's G_1_ rate model (Castor 1980) and Cooper's continuum model (Cooper 1982) can be adapted to recreate all of the desired properties listed above. While other models might be possible, we present here a detailed elaboration of a modified rate model to illustrate a projected underlying biological mechanism.

### 2.7. A modified rate-based model

**Figure 6.**
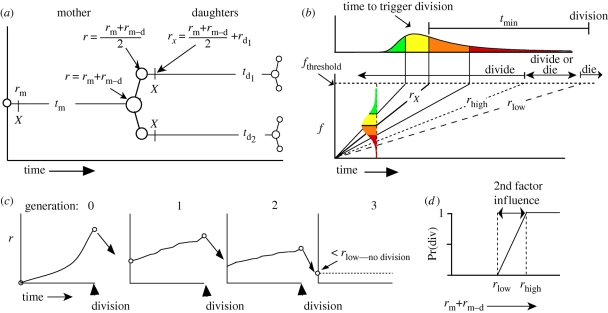
(*a*) Time line showing mechanism for inheritance of division time. Division time is determined by the value of *r*, shown here as *r*_*X*_, at the times marked *X*. For the mother cell, this is *r*_m_, and so its division time is given by 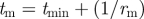. Between this time and division occurring, the mother cell increases its value of *r* by an amount, *r*_m−d_. At division, the total value of *r*, *r*_m_ + *r*_m−d_, is assumed to be split evenly between the daughter cells. Some time between division and the point marked *X*, each daughter cell acquires a further independent contribution to *r* called 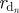, where *n* = 1, 2. As with the mother cell, the daughters now have division times determined by their value of *r* at *X*, which is now 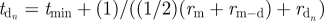. (*b*) Right skewed distribution arises from a rate-and-comparator model for replication. The quantity *f* is accumulated according to *f* (*t*) = *mrt*, where *m* is some constant of proportionality with units of *f* and *r* is a rate with units inverse time. Upon reaching a threshold value, *f*_threshold_, division machinery is initiated. At this point, 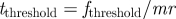 and so there is an inverse relationship between the value of *r* and the variable component of division time, *t*_threshold_. If *r* is distributed normally according to *N*(*r*) as shown in the vertically oriented distribution, then *t*_threshold_ is distributed with an inverse-normal pdf, drawn horizontally. The shaded areas show a cohort of cells with a particular range of values *r* and *t*_threshold_. (*c*) The value of *r* at first division is high and so nearly all cells divide. As cells go through consecutive division cycles, the quantity *r* gradually diminishes until they are unable to divide further. (*d*) Mid-response, the value of *r* passes through a narrow region in which stochastic fate selection can occur. Here, a second factor controlling cell death is also able to influence the probability of division.

The G_1_ rate model (Castor 1980) introduced the idea that the time taken for sibling cells to pass through G_1_ is correlated and that this can be modelled by distributing the rate of passage as a bivariate normal distribution. Our reason for adopting this distribution, as will be revealed, is that it reproduces the observation that siblings that divide early are more highly correlated than those that divide late. Our approach will be to generalize the distribution so that it can be applied beyond sibling correlations and explain correlations between different generations of cells. Castor's model also contains a stochastic mechanism to explain the passage of cells through a second (S + G_2_ + M) phase. We find this to be unnecessary for our purposes and replace it with a fixed time which we call *t*_min_. Hence, we write the following form to describe the division time of a B lymphocyte:
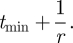



If *r* is distributed normally and with a positive lower bound (justified below on physical grounds), then this gives a right skewed distribution with some minimum division time, *t*_min_, assumed to be constant for all cells. The correlation between time to divide and propensity to divide suggests that the quantity *r* is somehow associated with the ability to enter into division. Consequently, we adopt a simple physical interpretation for *r* due to Cooper (1982) and postulate that *r* is proportional to the rate of synthesis of an initiating factor within each cell which, upon reaching a threshold level, triggers initiation of cell division (figure 6*b*). Events subsequent to this trigger can be thought of as taking time *t*_min_. If the concentration of this initiating factor is *f*, then we can write
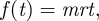

where *m* is a constant that converts to units of concentration and *t* is the time since division. For the purpose of notational convenience in the equations and discussion that follow, we set *m* = 1 and refer only to *r*, which has units of inverse time. As *r* represents a rate of synthesis, it must be positive and as the observed *t* has an upper bound *r* must be bounded from below. We speculate that *r* corresponds to an ensemble of contributing elements such as enzymes involved in signalling cascades and transcription factors regulating expression of essential proteins for growth. While the contributions from such elements may fluctuate over the course of the cell cycle, in order to keep our model as simple as possible, we take *r* to be constant during the accumulation of the initiating factor, *f*, but allow it to fluctuate at other times (i.e. between when *f* initiates division and when division actually occurs).

In order to introduce the correlations between the division times of two siblings and between the mother and daughter cells, we must describe the manner in which each new cell takes on its value of *r*. It is clear from our conclusions above that there is a degree of sharing between siblings that is inherited from the mother, and that the inherited level is predominantly dictating division times of the two siblings, given the high level of correlation. We note that there is less correlation between mothers and daughters than there is between siblings, suggesting that subsequent to division time being decided upon, but prior to division occurring, the value of *r* in the mother undergoes fluctuations which are then passed on to both daughter cells. This is in contrast to fluctuations in *r* that occur subsequently in each daughter cell which contribute to differences in sibling division times. The process is illustrated in figure 6*a*. The point in the cell cycle where division time is decided is marked with an *X*. Fluctuations in *r* beyond this point do not affect division time for the cell, only its daughters. The value of *r* available at *X* is equal to the sum of three parts: (i) the amount that was available to the mother at *X*, *r*_m_ (divided in two as it is split equally between daughters), (ii) variations in *r* that occurred in the mother after this point, *r*_m−d_ (also divided in two), and (iii) independent variation in *r* in each daughter cell up to this point, 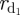 and 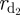. We can write this formally as follows:
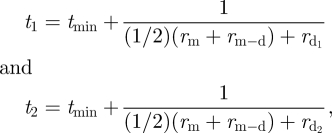

where *t*_1_ and *t*_2_ are the division times for sibling cells and *r*_m_ is the portion of *r* that contributed to the division time of the mother cell. That is, 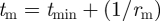, where *t*_m_ is the division time for the mother cell and *r*_m_ is responsible for the inherited component of division time as it contributes to the division times of both mother and daughters.

*r*_m−d_ is a normally distributed random number, 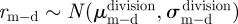. It gives the difference between mother and daughters which is common to both siblings. 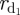 and 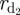 are normally distributed random numbers with zero mean, generated independently for each cell according to 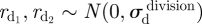. These are responsible for the difference between sibling division times.

For the initial generation of cells, we choose *r*_m_ to be normally distributed according to 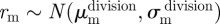. On rare occasions, total *r* can be very small or negative, resulting in unphysical division times. To prevent this, the distribution is truncated (see the electronic supplementary material for details).

We observe that cells with a large *r*_m_ + *r*_m−d_ tend to divide earlier. For such cells, the noise from 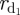 and 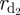 will be proportionately less, hence they will be more correlated, satisfying our initial criterion that siblings dividing earlier be more highly correlated. The fact that siblings are more correlated than mother–daughter pairs suggests that 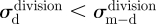 (table 1). In other words, most of the noise on *r* (and hence the physical quantity that it represents) is picked up between when division time is decided and when division actually occurs. One can speculate on the source of the noise, but suffice it to say that, if the physical quantities that determine *r* are produced and subject to imperfect regulation, then one would expect it to accumulate fluctuations over time (Sigal *et al*. 2006).

**Table 1.** Quantities used to generate simulated data from multivariate model.

parameter	value	parameter	value
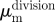	0.9	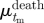	40
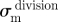	0.02	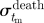	15
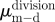	−0.03	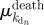	0.9
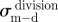	0.04	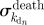	0.3
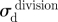	0.02	*t*_threshold_	10
*r*_high_	1/12	*t*_max_	25
*r*_low_	1/22	*n*_initialcells_	40
*t*_fixed_	4		

If 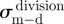 and 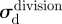 are tuned to give agreement with correlations between mother–daughter and sibling–sibling correlations, then we can predict the observed cousin–cousin correlations (figure 3*d*). This demonstrates that the model for division times captures salient features of the data.

### 2.8. Modelling death times

The above modified rate-based model recreated the key correlations in division times transmitted through generations. As noted, correlations, albeit weaker, are also found in death times through generations. Using the same approach, we seek the simplest model that can reproduce the key features of the data relating to the inheritance of death times. These features are as follows.

— Right skewed distribution.
— A trend to decreasing *t*_die_ in later generations.
— Correlated *t*_die_ for siblings and inheritance as demonstrated by correlation of cousin death times.
— Unlike *t*_divide_, *t*_die_ is not strongly correlated at small values.
— *t*_divide_ is independent of *t*_die_. While figure 5*a*,*b* shows some correlation between division and death times, the magnitude is small and the signs are conflicting. This suggests that the two may be modelled as independent processes.

Here again, we postulate that components making up the survival machinery of the cell are partly inherited from the mother, and partly made independently and de novo, in each new cell. We propose as the simplest case, and in absence of further information, that the quantity of the factor controlling time taken to apoptosis is directly proportional to the time to die of the cell. Thus, we chose a minimal generalization of the cyton model mechanism for death (Hawkins *et al*. 2007)
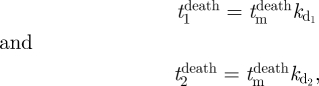

where 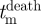 is the death time carried by the mother cell, which we identify as proportional to the level of our second common factor. Clearly, if this was passed on, the mother did not die. For undivided cells, we assume a lognormal distribution in the population of the factor and, therefore, lognormally distributed times to die as advocated in Hawkins *et al*. (2007), that is
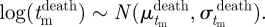


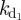
 and 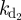 are components which produce independent variations for each daughter and are distributed according to 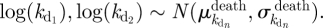


The parameter 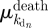 was chosen to give the correct trend in death times with generation while 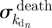 was selected to match the correlation between siblings that is observed in the data (figure 3*e*). In contrast to division times, we note that we require no analogue to *r*_m−d_, suggesting that, for time to die, all of the variation in *t*_die_ subsequent to division arises independently in the daughter cells.

### 2.9. Linking fate determination to division time

In developing a model to explain the connection between fate determination and division time, we need to keep in mind the growth data from Hawkins *et al.* (2009), which suggest that cell fate is determined at, or soon after, cell division. The early determination of cell fate, plus the high correlation of cell fate as previously described here, suggests that perhaps only inherited material from the mother, *r*_m_ + *r*_m−d_, need be used. Even so, approximately 10 per cent of cells undergo different fates, so a further random component specific to each sibling is required. One option would be to include 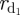 and 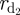, that is, select cell fate based on the total value of *r*. But this would lead to a sharp cut-off in the distributions for division time, which is something the data do not support. We propose that a stochastic process acts on cells to produce uneven fates. Looking at the division times for cells whose sibling died (Hawkins *et al.* 2009) suggests that this mechanism acts only on cells with long division times, that is, when cells are on the cusp of being able to divide. To summarize, if 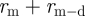 is more than *r*_high_, then both siblings divide. If *r*_m_ + *r*_m−d_ is less than *r*_low_, then both siblings die. If *r*_m_ + *r*_m−d_ is in between *r*_low_ and *r*_high_, then each cell has a stochastic outcome dependent on the value of *r* that it has associated with it. In this intermediate region, we propose that the probability of division increases linearly with *r*_m_ + *r*_m−d_ to effect a smooth transition. Thus, we model the probability of division of each cell as
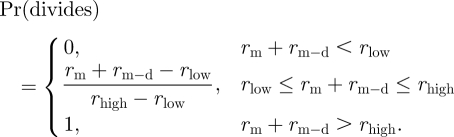



Figure S1*b*,*c* in the electronic supplementary material shows that this model gives the correct qualitative relationship between propensity to divide and *t*_divide_. Furthermore, because only the inherited component and not the individual components of *r* (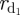 and 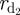) are used to decide whether a cell can divide, the propensity of sisters to divide can have a similar correlation to time to divide, as is observed in the data. Because only half of *r* is passed on to the daughter cells at division and because *r*_m−d_ can, on average, be negative, the average *r* of a population of cells is depleted over successive generations. As this depletion occurs, the distribution of *r* for the overall cell population will pass through the region between *r*_low_ and *r*_high_. As it does so, the proportion of cells dividing and the correlation between fates for sisters both have the correct qualitative form. That is, early in the response most cells divide; uneven cell fate is most likely to occur mid-response; and late in the response, most cells die (figure 6*c*,*d*). Finally, the distribution of *r* in the founder population and the subsequent preservation of relative levels in descendents that arises from the proposed mechanism leads to the strong founder effect for division destiny (Hawkins *et al.* 2009).

### 2.10. Linking fate determination to death time

The model has many of the features sought; however, as it presently stands, it will not show any dependence between times to die and propensity to divide as shown in figure 4. One reasonable way to include this is to make the probability of division dependent on a functional combination of *t*_divide_ and *t*_die_ (or components thereof). Unfortunately, this tends to lead to unphysical *t*_divide_ and *t*_die_. Removing such unphysical subpopulations by fiat leads to unintended correlations between the two times (data not shown). To avoid these two problems, we instead make a minor change to the stochastic cut-off procedure above to incorporate the inherited component of death time, 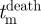. The fate of cells with intermediate values of *r*_m_ + *r*_m−d_, that is, where *r*_low_ < *r*_m_ + *r*_m−d_ < *r*_high_, now depends on the value of this second common factor. Cells with this intermediate amount of *r* can be thought of as being on the cusp of being assigned to either death or division. Such cells are sensitive to a second signal to decide cell fate; a signal (in the form of a high 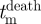) that causes both of them to die. Alternatively, if such cells are not sufficiently committed to death (by having a low 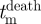), then they are assigned different fates as before. Formally,
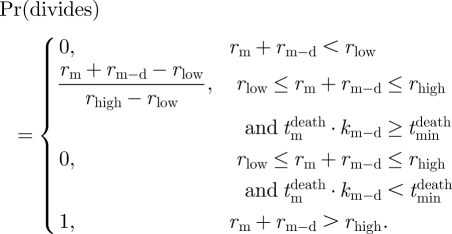



We find that setting
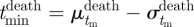

gives us the correct qualitative dependence of time to die on propensity to divide (electronic supplementary material, figure S1*a*) while preserving the dependence of time to divide on propensity to divide (electronic supplementary material, figure S1*b*,*c*). It also points to the existence of a subpopulation of cells on the verge of not being able to progress further through divisions, which are sensitized to other signals to trigger fate selection.

## 3. Discussion

The recent data of Hawkins *et al*. (2009) recorded the correlation in times to divide and die in B-cells following stimulation with CpG that leads to proliferation, eventual cessation after a varying number of division rounds and then death. Striking familial correlations were observed that we reasoned could provide a unique insight into the source of shared and randomized components of cell fate in this system. To facilitate this goal, we sought a minimal model that could reproduce the important features of the data. We found that a minimum of two different heritable factors was necessary to explain the correlation structure in the data. We then showed that two heritable factors were sufficient by constructing a model postulating one heritable factor that controls a cell's time to divide and another which regulates time to apoptosis. In our model, both factors play a role in determining cell fate. We find the data are consistent with a mechanism where the value of the first factor varies the rate of accumulation of downstream cellular mediators(s) that trigger cell division when a threshold is reached. Stochastic variation in parts of this mechanism, either in the level of the inherited factor, the level of accumulated mediators or in the selection of the threshold level for triggering division, contributes to deciding cell fate (either division or death). While we make no presumptions about the particular physical mechanism involved, if we assume that the second factor also modulates this stochastic fate selection, then we can correctly describe the regulation and correlation between all three aspects of cell number regulation without the need for any further heritable components.

One of the striking features of the Hawkins *et al*. (2009) data is the extreme correlation of propensity to divide. On average, this is more strongly correlated than any other quantity between siblings. In order for propensity to divide to be more strongly correlated than time to divide, it had to be derived from the common component influencing time to divide inherited from the mother cell. This feature did not include the additional random component of time to divide that contributed independently to division times of each sibling. In other words, time to divide picked up more randomness at or after division whereas division and propensity to divide did not. This is consistent with the hypothesis that cell fate is decided at, or soon after, division, as was suggested by examination of cell size in Hawkins *et al.* (2009).

The tracking of cell lineages was undertaken by Hawkins *et al*. (2009) to highlight the source and nature of variation in lymphocyte regulation. Numerous prior models have been proposed to describe lymphocyte proliferation and survival, although few accommodate such strong lineage affiliations as revealed in this new dataset. Furthermore, most models interleave cell division and death by assuming an age-independent time to die that is inconsistent with the pattern of death observed in these data. An earlier paper by Hawkins *et al*. (2007) proposed the cyton model based on the hypothesis that times to divide and die were independent, and acted in competition, with each being clocked from their last division and following some skewed right probability. Here, our minimal model also assumes that division and death are clocked from mitosis. However, in contrast to the cyton model, we assume cells decide their fate, either division or death, early after a division, and that time to the chosen fate is then regulated. This simpler model is possible because we recorded no instance of cells dying during a growth phase leading to a cell division. Rather, only cells that lost the impetus to grow went on to die. The cyton model is capable of reproducing this behaviour by having a distinct time to die distribution for cells that have undergone division destiny (Subramanian *et al*. 2008). The generality of this and other methods for incorporating death in useful biologically relevant mechanisms will become apparent only when additional regulated cell systems are followed in a similar manner to the CpG-stimulated B-cells studied here.

We do not rule out the possibility that there may exist other common factors than the ones proposed. Nor do we rule out the possibility that a model with more degrees of freedom might give outcomes that agree more closely with observations. For example, the model could be extended to give better agreement with correlations between distantly related cells in a pedigree. However, in order to explain a subset of the observed correlations in our data, we already require a significant number of degrees of freedom; each correlation needs to be parameterized, as does division-linked behaviour. The decision on how far to go down the path of increasing model complexity to fit data is based on whether doing so adds insight into the system or utility. In this case, formulating a model to describe the operation of two common factors leads to the discovery that one of the common factors acted only on cells that were sensitized to respond. That is, the common factor for death could only affect a subset of the total cell population. Beyond the insight gained, a question that remains unanswered is ‘do we need to use these multivariate models to analyse population experiments?’ In responses that are limited to relatively few division cycles, existing univariate models are sufficient for the purposes of reproducing the mean population sizes. However, as the number of division cycles increases, the effect of correlations in division time between parents and their progeny on the mean population dynamics increases, and it becomes necessary to use a model that accounts for this correlation (Wellard *et al*. submitted). Furthermore, studies using branching process analyses (Crump & Mode 1969; Duffy & Subramanian 2009; Wellard *et al*. submitted) suggest that correlation in time to divide and in cell fate necessarily leads to increased variability of total cell numbers. For simulation of small populations of cells, this can have an impact on the results. For example, fluctuations in the numbers of a small clone of lymphocytes could result in its extinction. A study using a model that incorrectly implements correlation between cell fates would be unable to capture this behaviour. The conclusion to be drawn from this is that the choice of model depends on the application. Utility would suggest that, for systems of thousands of cells, the simplest univariate model as measured by ease of solution and goodness of fit should be used. For clones of tens of cells, as can exist at the beginning of an immune response, the model needs to capture correlated behaviour and the solution method needs to be able to calculate the fluctuations about the expected mean behaviour.

Finally, our modelling approach leads to testable biological hypotheses and suggests directions for future investigation. Our study suggests that it would be fruitful to search for cell surface, cytoplasmic or nuclear proteins diluting and varying from generation to generation that are involved in triggering both division and death. This could be achieved by proteomic analysis, which makes no assumptions, or a candidate investigation of probable cell cycle and cell death regulators. As the expression of putative factors appears to be required primarily in the first division, and less so in subsequent divisions, high throughput sequencing of RNA (Mortazavi *et al*. 2008) might be used to compare RNA expression levels for cohorts of cells from consecutive divisions to identify candidate transcripts with these features. Once the diluting elements controlling the division and death times are identified, expression as fluorescent-tagged fusion molecules would allow further time-lapse microscopy experiments to correlate and monitor the stochastic inheritance and re-synthesis in each generation predicted here. The existence of heritable factors regulating the propensity to divide with times to divide and die raises the prospect that there may exist more heritable factors, changing with division, that regulate other aspects of the immune response. It has been shown that differentiation decisions for both B lymphocytes and T lymphocytes alter with successive division rounds (Hodgkin *et al*. 1996; Bird *et al*. 1998; Gett & Hodgkin 1998). We speculate that a similar quantitative approach applied to following alternative fates may be able to provide further insight into regulatory mechanisms of the immune response and the control of the rapid emergence of cellular heterogeneity.
